# Contraceptive use in women with mental illness in Soweto, South Africa

**DOI:** 10.4102/sajpsychiatry.v30i0.2153

**Published:** 2024-01-25

**Authors:** Lisa J. Galvin, Yvette M. Nel

**Affiliations:** 1 Department of Psychiatry, Faculty of Health Sciences, University of the Witwatersrand, Johannesburg, South Africa; 2 Perinatal HIV Research Unit, Wits Health Consortium, University of the Witwatersrand, Johannesburg, South Africa

**Keywords:** mental health, mental illness, contraception, women, South Africa

## Abstract

**Background:**

The psychosocial and medical implications of unplanned pregnancy in women with mental illness (MI) are vast. International guidelines make clear recommendations about family planning for women with MI, particularly those exposed to known human teratogens; however, there is limited research related to contraceptive usage among women with MI.

**Aim:**

The aim of this study was to investigate the prevalence of consistent contraceptive use and family planning education (FPE) among a population of women of childbearing age with MI.

**Setting:**

This quantitative cross-sectional study was conducted at Chris Hani Baragwanath psychiatric unit in Soweto, South Africa.

**Methods:**

A convenience sample comprising 190 eligible women of childbearing age with MI was employed for the study. The women were invited to participate by means of a structured questionnaire which was administered by the researcher. Clinical information was obtained from the patients’ medical records.

**Results:**

Consistent contraceptive usage occurred in 44.7% of participants. Family planning education was low (26.8%). Relationship status was associated with using contraception consistently (*p* = 0.0229). Teratogen exposure was not associated with either contraceptive use or FPE. Family planning education was not associated with contraceptive use.

**Conclusion:**

Women with MI may have increased risk for unplanned pregnancy if they are not in a relationship because of perceived lack of need for contraception.

**Contribution:**

Family planning education must be prioritised in women with MI, especially among women prescribed teratogenic medication, highlighting the risks associated with unplanned pregnancy.

## Introduction

Widespread access to family planning and contraceptives choices for women of childbearing age are limited, particularly in low- to middle-income countries.^1^ Women with mental illness (MI) are at increased risk of unplanned pregnancy and contracting sexually transmitted infections (STIs).^2,3^ Women with MI are also at increased risk of gender-based violence and sexual coercion^4^ which may further increase the risk of unplanned pregnancy and contracting STIs. Despite this added risk, women with MI often receive inadequate family planning education (FPE) and reproductive health support.^5^ Although motherhood is often a positive experience and may provide persons living with MI with an increased purpose in life and motivation to be well, women with MI have, on average, higher rates of unemployment, poverty, and weak social support^6^ increasing the burden of childcare.

Unplanned pregnancy is a risk factor for MI^7^ as well as increasing risk of psychosocial stressors, further increasing the risk of poor mental health.^8^ The added risk of poor social support, poor socioeconomic status, and domestic violence associated with being a woman living with MI thus compounds the already increased risk of poor mental health when a pregnancy is unplanned.^4,6,8^

Little is known about contraceptive choices and barriers and facilitators to contraceptive use in women with MI. Half of the women with MI in Turkey used contraception, with coitus interruptus being the commonest method and less than 15% of women using barrier methods.^9^ Low levels of contraceptive knowledge influenced contraceptive choice in women with schizophrenia in Turkey.^10^ In Ethiopia, half the women with MI used contraception, with 29.7% using oral hormonal contraception, 26.7% using injectables, and 22% using condoms.^11^ Fears of drug interactions as well as a lack of knowledge influenced contraception use.^11^ Barriers and facilitators to contraceptive use in women and contraceptive preferences in women with MI in the South African context are unknown.

Unplanned pregnancy is prevalent in South Africa, with half of pregnancies being unplanned.^12^ The South African Demographic and Health Survey of 2016 (SADHS 2016) reported that 55% of South African women used contraception.^13^ There is little knowledge regarding unplanned pregnancy in women with MI in South Africa; however, Du Toit et al. reported in 2018 that almost half of women attending specialised maternal mental health clinics in Cape Town had unplanned pregnancies.^14^ Prevalence of contraceptive use by women with MI in South Africa is unknown. In women with MI attending outpatient services in Kenya, contraceptive prevalence was 42.2% and only 34.3% had received FPE.^15^ In Nigeria, 88% of women with MI knew at least one method of contraception but only 27% used contraception and 5% had received FPE.^16^

Pregnancy, and the post-partum period, is a period of increased risk in women with MI, with increased risk of relapse and hospitalisation in conditions such as bipolar disorder and depression.^17^ Children born to women with MI are at increased risk of physical and mental adverse health outcomes.^18,19^ Exposure to teratogenic medication, such as sodium valproate, which is associated with congenital abnormalities, is a well-documented risk in women of childbearing age who are prescribed such teratogens without adequate FPE and contraceptive cover.^20^ Family planning education is needed in women with MI to ensure that they are aware of their specific risk of adverse outcomes if they fall pregnant in order to make informed choices about their reproductive health. If pregnancy is well-planned, women may be able to reduce teratogen exposure and optimise safe exposure to relapse preventative medication during pregnancy. They may also be better equipped to optimise social support structures to enhance mental health and well-being during pregnancy and the postpartum period if pregnancy is planned, thereby improving outcomes for both mother and child.

The need for family planning services in women with MI is evident, yet there is a lack of literature examining the frequency of FPE provided to women and the prevalence of contraceptive use in women with MI in South Africa. Contraceptive preferences and barriers and facilitators to accessing contraception in women with MI need to first be defined before family planning needs can be met. The aim of this study was to assess contraceptive use, as well as FPE exposure, in women attending mental health services in Soweto, South Africa.

### Objectives

1. To determine the prevalence of contraceptive use in women with MI attending Chris Hani Baragwanath Academic Hospital (CHBAH).
2. To determine the prevalence of FPE by mental health care providers (MHCPs) in women with MI attending CHBAH.
3. To determine the demographic and clinical factors associated with contraceptive use and FPE exposure in women with MI attending CHBAH undergoing routine psychiatric follow-up.

## Research methods and design

### Study design and setting

This quantitative cross-sectional study was conducted at CHBAH in Soweto, South Africa from 03 October 2016 to 28 April 2017. A convenience sample comprising 190 eligible women of childbearing age with MI from the CHBAH Psychiatric Outpatient Clinic and from inpatients in the psychiatric wards was obtained. Inpatients were screened once they were psychiatrically stabilised and able to provide informed consent. Childbearing age was defined as 18–49 years of age. This range was selected as 18 is not only the cut-off age for informed consent but also because women with MI below the age of 18 years are treated by the child and adolescent psychiatry unit. Literature suggests a cut-off of 49 years of age when defining childbearing age.^15,16^

Women were excluded from this study if they did not have the capacity to consent to participation. They were also excluded if they were post-menopausal prior to the age of 49 years.

**FIGURE 1:**
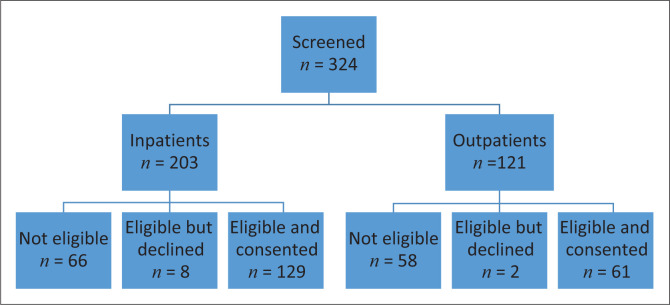
Screening of patients for inclusion in study.

A total of 324 patients were screened until the sample size of 190 was obtained (Figure 1).

### Data collection

Demographic characteristics of participants and self-reported contraceptive use, as well as information regarding FPE were acquired through a clinician administered questionnaire. Copies of the questionnaire, informed consent, and distress protocol were available in English and Zulu. The questionnaire was piloted on 10 participants and revised accordingly. Contraceptive use was defined as self-reported use of contraception at the time of data collection. Relationship status was defined as single if participants reported being single, divorced, widowed, or separated, while being in a relationship was defined as being married, engaged, or in a relationship.

Contraception was defined as follows:

- Exogenous hormonal contraception: Depot and oral hormonal contraception. Endogenous hormonal changes occurring naturally during the menstrual cycle (rhythm method) or during breastfeeding were not included. Emergency oral contraception was not included because it is not recommended for regular contraception.
- Non-hormonal contraception: Permanent methods (surgical sterilisation by means of tubal ligation or hysterectomy), barrier methods (male and female condom, spermicides), natural methods (coitus interruptus, rhythm methods and breast feeding), and intrauterine devices (IUDs). Intrauterine devices were included in non-hormonal contraception because participants were unable to specify which form they were using and copper IUDs are more commonly used than hormonal IUDs in the government health care setting in Soweto, South Africa.

Consistency of use was defined as contraception use during every instance of sexual activity as opposed to ‘sometimes’ as contraception needs to be used during all instances of sexual activity for it to be effective.

Teratogen use was defined as the use of any of the following: valproate, lithium, carbamazepine, or lamotrigine. Diagnosis according to the Diagnostic and Statistical Manual (DSM-V) diagnostic criteria^21^ and medication was obtained from patient files. Family planning education was defined as any advice received around pregnancy planning given by a MHCP.

### Ethical considerations

Ethical clearance was obtained from the Human Research Ethics Committee of the University of the Witwatersrand (M160433). Permission was also obtained from the CHBAH research committee. Informed consent was obtained prior to completion of the questionnaire or access to patient records. Patient information sheets were available in English and Zulu. A distress protocol was in place to assist participants reporting intimate partner violence or other abuse. A referral system was also in place for participants requiring more information on family planning. Patient confidentiality was maintained as no identifying information was recorded.

### Data analysis

Data were entered in Excel and all statistical analysis was conducted in SAS Enterprise Guide 7.1 (SAS Institute, Cary, NC). The 5% significance level was used. Descriptive analysis of the data was performed. The *Χ*^2^ test was used to determine association between categorical demographic and clinical variables and presence or absence of regular contraceptive use and presence or absence of FPE. Fisher’s exact test was used for 2 × 2 tables or when the requirements for the *Χ*^2^ test could not be met. The Wilcoxon Rank Sum test was used for continuous variables as data were non-parametrically distributed.

## Results

Median age of the total sample was 31 years (IQR: 26–39; range 18–49). Two thirds of participants were single (66.3%, *n* = 126) and 44.2% (*n* = 84) were sexually active and 42.6% (*n* = 81) planned to have children in the future. Most participants (48.9%, *n* = 93) had not completed high school, with 28.9% (*n* = 55) having attained matric, and 22% (*n* = 42) having a tertiary education. Only 17.9% (*n* = 34) of participants were employed. Most participants were Christian (77.4%, *n* = 147) (Table 1). There were no significant differences in demographic characteristics between inpatients and outpatients.

**TABLE 1:** Comparison of demographic characteristics between participants using contraception and participants not using contraception.

Variable	Overall (*N* = 190)				Consistent contraception (*N* = 85)				No consistent contraception (*N* = 105)				*P*
	*n*	%	Median	IQR	*n*	%	Median	IQR	*n*	%	Median	IQR	
**Age**	-	-	31	26–39	-	-	31	27–38	-	-	31	26–39	0.622
**Relationship status**													
In a relationship	64	33.7	-	-	36	42.4	-	-	28	26.7	-	-	0.023*
Single	126	66.3	-	-	49	57.6	-	-	77	73.3	-	-	
**Employed**	34	17.9	-	-	14	16.5	-	-	20	19.1	-	-	0.645
**Highest level of education**													
Incomplete high	93	49.0	-	-	43	50.6	-	-	50	47.6	-	-	0.870
Matric	55	28.95	-	-	23	27.06	-	-	32	30.48	-	-	
Tertiary	42	22.1	-	-	19	22.4	-	-	23	21.9	-	-	
**Religion**													
Christian	147	77.4	-	-	72	84.7	-	-	75	71.4	-	-	0.130†
Traditional African	22	11.6	-	-	6	7.1	-	-	16	15.2	-	-	
Muslim/JW/Buddhist	12	6.3	-	-	5	5.9	-	-	7	6.7	-	-	
None/Unsure	9	4.7	-	-	2	2.3	-	-	7	6.7	-	-	
**Desires future children**	81	42.6	-	-	31	36.5	-	-	50	47.6	-	-	0.122
**Sexually active**													
Yes	84	44.2	-	-	43	50.6	-	-	41	39.1	-	-	0.111
No	106	55.8	-	-	42	49.4	-	-	64	60.9	-	-	
**Family planning advice given (yes)**	51	26.8	-	-	22	25.9	-	-	29	27.6	-	-	0.788

*P* value

* = statistically significant (*p* ≤ 0.05);

† = size of group too small to perform statistical analysis (only Christian and Traditional African religion compared).

The commonest diagnoses were bipolar affective disorder (49.5%, *n* = 94), depression (30.2%, *n* = 57), and psychotic disorders (23.2%, *n* = 44). The commonest medication classes were antipsychotics (71.4%, *n* = 135) followed by mood stabilisers (45.5%, *n* = 86) and antidepressants (36%, *n* = 68) (Table 2). There was missing data on diagnosis and medication for one patient which was erroneously not recorded.

**TABLE 2:** Comparison of clinical characteristics between participants using contraception and participants not using contraception.

Variable	Overall (*N* = 189)†		Consistent contraception (*N* = 84)		No consistent contraception (*N* = 105)		*P*
	*n*	%	*n*	%	*n*	%	
**Diagnosis (yes)**							
Bipolar	94	49.5	36	49.2	58	55.2	0.110
Depressive	57	30.2	34	40.5	23	21.9	0.006*
Psychotic	44	23.3	19	22.6	25	23.8	0.847
Personality	25	13.2	13	15.5	12	11.4	0.414
Substance	17	9.0	6	7.1	11	10.5	0.426
Cognitive disorders	8	4.2	5	6.0	3	2.9	0.470
**Prescribed psychotropic medications**							
Antipsychotic	135	71.4	58	69.0	77	73.3	0.517
Mood stabiliser	86	45.5	37	44.0	49	46.7	0.719
Antidepressant	68	36.0	35	41.7	33	31.4	0.145
**Teratogen (*n* = 189)**	79	41.8	33	39.3	46	43.8	0.531
Valproate	68	36.0	28	33.3	40	38.1	0.54
Lithium	10	5.3	4	4.8	6	5.7	> 0.99
Lamotrigine	14	7.4	9	10.7	5	4.8	0.16
Carbamazepine	3	1.6	0	0	3	2.9	0.26

*P* value

* = statistically significant (*p* ≤ 0.05);

† = *N* = 189 as one participant had missing data.

### Prevalence of contraceptive use in women with mental illness

Contraception was used ‘always’ or ‘sometimes’ by 60% (114 of 190 participants); however, only 44.7% of participants (85 of 190 participants) reported using contraception consistently (‘always’). Exogenous hormonal contraception was used by 27.9% (*n* = 53) of the total study population (45.6% of participants using contraception). Non-hormonal contraception was used by 38.9% (*n* = 74) of the study population (64.9% of participants using contraception). Non-hormonal and exogenous hormonal contraceptive use do not sum to 100%, as some participants used more than one method.

**FIGURE 2:**
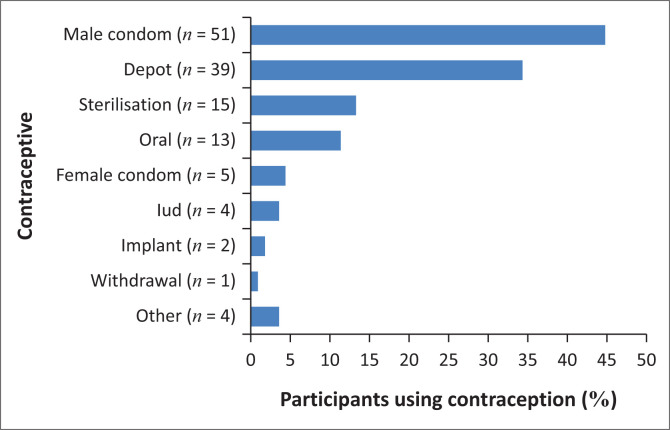
Individual methods of contraception displayed as a percentage of total number of participants using contraception (*n* = 114).

Barrier methods of contraception were the most popular choice (28.9% of total study population, *n* = 55) (Figure 2). Of 55 women using barrier methods, 51 used male condoms and 5 used female condoms. One woman used both methods. No women used spermicides. Depot hormonal contraception was the second most popular choice (20.5% of study population, *n* = 39), while 6.8% (*n* = 13) of women used oral hormonal contraception (Figure 2). Permanent methods of contraception (sterilisation) were used by 7.9% (*n* = 15) of women, and three women (1.6% of total population) used natural methods of contraception (one used coitus interruptus and two women used breast feeding) (Figure 2).

### Prevalence of family planning education by mental health care providers in women with mental illness

Of the 190 participants, 26.8% (*n* = 51) had received FPE. Family planning education was not associated with contraception use as 43.1% of women (*n* = 21) who had received FPE used contraception compared to 45.3% (*n* = 63) who had not received FPE (Chi-square test, *p* = 0.788).

### Demographic and clinical factors associated with contraceptive use and family planning education exposure in women with mental illness

#### Demographic factors

Relationship status was the only demographic characteristic associated with contraceptive use (Table 1). Single women were less likely than women in a relationship to use contraception (Fisher’s exact test, *p* = 0.023) (Table 1). Being single did not equate to sexual abstinence as 11% (*n* = 20) of women reported being single but still sexually active. Sexual activity was not associated with contraceptive use (Fisher’s exact test, *p* = 0.111) (Table 1).

Women using exogenous hormonal contraception were younger than those who used non-hormonal contraception (Wilcoxon rank sum test, *p* = 0.0031). The age of women using hormonal contraception ranged from 20 to 45 years with a median (interquartile range) of 28 years (24–34 years of age) compared to those not using hormonal contraception and whose age ranged from 18 to 49 years with a median (interquartile range) of 33 years (28–41 years of age). There were no other associations between the demographic characteristics of women using contraception and use of exogenous hormonal or non-hormonal contraception use.

Relationship status and barrier contraception use were associated with 57.6% of single contraceptive users using barrier contraception (*n* = 38) compared to 35.4% of contraceptive users in a relationship (*n* = 17) (Fisher’s exact test, *p* = 0.023).

While there was no association between contraception use and level of education (Fisher’s exact test, *p* = 0.870), a higher level of education was associated with increased use of barrier methods, with 68.2% (*n* = 15) of women with tertiary education, 53.3% (*n* = 16) of women with matric, and 38.7% (*n* = 24) of women who had not completed high school using barrier contraception. (Fisher’s exact test, *p* = 0.048).

Median age of participants who used permanent methods was older than those who used other methods of contraception (Wilcoxon rank sum test, *p* = 0.0001). The age of women using permanent contraception ranged from 28 to 49 years with a median (interquartile range) of 43 years (34–46 years of age) compared to those not using permanent contraception whose age ranged from 18 to 49 years with a median (interquartile range) of 29 years (25–35 years of age). Women using permanent methods of contraception were less likely to desire future children (6.7%, 1 out of 15 women) compared to women using other methods of contraception (41.4%, 41 of 99 women) (Fisher’s exact test, *p* = 0.013). There was no association between religion and contraception use (Fisher’s exact test, *p* = 0.068).

More women who had received FPE (31.4%, *n* = 16) were employed compared to those who had not received FPE (12.6%, *n* = 18) (Fisher’s exact test, *p* = 0.015). Women using oral hormonal contraception were more likely to have received FPE (61.5%, or 8 of the 13 women) compared to women who used other forms of contraception (25.7%, or 26 of the 101 women) (Fisher’s exact test, *p* = 0.020).

#### Clinical characteristics

More women with depression used contraception compared to those without depression (Fisher’s exact test, *p* = 0.006) (Table 2). There was no association between psychiatric medication choice and contraception use (Table 2). There was, however, an association between medication class and specific contraceptive choice. Of the 53 women using contraception who took mood stabilisers, 75.5% (*n* = 40) chose non-hormonal contraception while 24.5% (*n* = 13) chose exogenous hormonal contraception (Fisher’s exact test, *p* = 0.048). E4. Teratogen use was not associated with contraceptive use (Fisher’s exact test, *p* = 0.531) (Table 2). Contraception was used by 41.2% (*n* = 28) of women using valproate; 64.3% (*n* = 9) of women using lamotrigine; 40% (*n* = 4) of women using lithium; and no women using carbamazepine used contraception. (Table 2).

There was no association between receiving FPE and medication class nor was there an association with FPE and diagnosis. Notably, 17.5% (*n* = 33) of women were using teratogens and desired future pregnancies and one woman was unsure if she wanted children. There was no association between teratogen use and receiving FPE (Fisher’s exact test, *p* = 0.740). Family planning education had been given to 28.0% (*n* = 19) of women using valproate; 35.7% (*n* = 5) of women using lamotrigine; 30% (*n* = 3) of women using lithium; and none of the women using carbamazepine.

**FIGURE 3:**
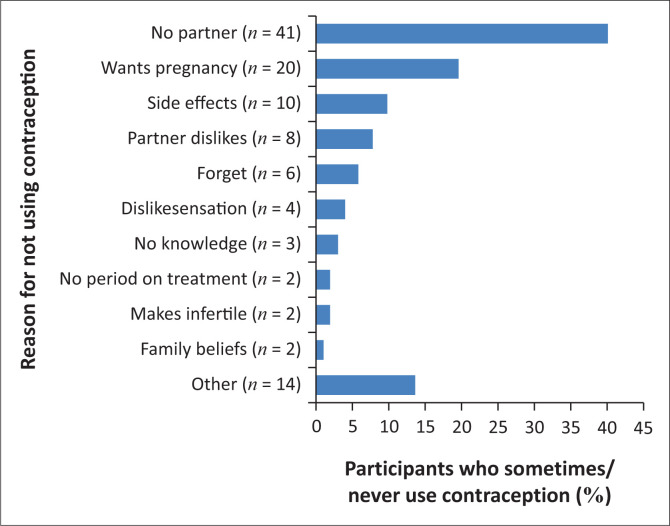
Reported reasons for not using consistent contraception (*n* = 105).

The commonest reason for not using contraception was not having a partner, accounting for 40.2% (*n* = 41) of women who did not use contraception (Figure 3). The second most common reason for non-use of contraception was the desire to fall pregnant, which accounted for 19.6% (*n* = 20) of women not using contraception (Figure 3). Desiring future children was, however, not associated with contraceptive use/non-use in the total sample (Fisher’s exact test) (*p* = 0.122) (Table 1).

## Discussion

### Contraceptive use in women with mental illness

Contraceptive use in women with MI in this study was similar to the general South African female population, with 60% (*n* = 114) of participants reporting any current contraception usage compared to 55% of women using contraception in SADHS (2016).^17^ When women were asked if they used contraception ‘always’ or consistently, this figure dropped to 44.7% (*n* = 85). This suggests that there may be awareness of the need for contraception and desire to use contraception in women with MI; however, this is not translating into regular consistent use. Although there is an increased risk of unplanned pregnancy in women with MI,^2,3^ and increased risks specific to women with MI (including but not limited to increased teratogen exposure,^20^ risk of MI relapse^17^ and psychosocial implications^8^), this did not translate to a higher frequency of contraceptive usage when compared to the general population.

### Demographic characteristics and contraceptive use

The only demographic characteristic associated with contraceptive use was relationship status, with single women being less likely to use contraception (Fisher’s exact test, *p* = 0.023). Similar associations with single relationship status and reduced contraceptive use have been found in Kenyan women with MI^15^ as well as the general South African population.^13^ This is important as almost two thirds (66.3%, *n* = 126) of women in the study reported being ‘single’. Women who are not in a relationship may not be using contraception as they may believe that they will not be at risk of falling pregnant as they do not have a regular sexual partner. This is supported by studies showing that being single is associated with unplanned pregnancy.^22^ Despite 33.7% (*n* = 64) of women in this study reporting being in a relationship, 44.2% (*n* = 84) of the participants reported being sexually active. Being single therefore did not equate to sexual abstinence. Women’s perceived need for contraception only if they were in a relationship suggests that women with MI may be vulnerable to unplanned pregnancy. This was substantiated by women reporting being single as the commonest reason for not using contraception. There was no association between being sexually active and contraception use (Fisher’s exact test, *p* = 0.11) (Table 1). Women who were single were more likely to use barrier methods of contraception. This may be because women in a relationship may have perceived their risk of contracting STIs to be lower as they had a regular sexual partner as opposed to women who were single. This study did not, however, explore perceptions around STIs.

Women who had a higher level of education were more likely to use barrier contraception. Studies have demonstrated that level of education is associated with increased condom use in South Africa, and women who have a higher level of education are more likely to be empowered to negotiate condom use.^23^

Younger women were more likely to use hormonal contraception than older women and women who did not desire pregnancy were more likely to use permanent methods of contraception. It is possible that as women age, medical concerns (e.g. cardiovascular risk or cancer) may cause contraindications to hormonal contraception and increase the need for permanent methods of contraception; however, this study did not record medical comorbidities nor did it differentiate between permanent methods chosen for medical illness as opposed to a means of contraception.

### Association between clinical characteristics and contraception

Women with depressive disorders were more likely to use contraception than those with other psychiatric diagnoses (Fisher’s exact test, *p* = 0.006). There was association between other psychiatric diagnosis and contraceptive utilisation. Studies have reported associations between hormonal contraceptive use and depression because of hormonal influences of contraception on mood; however, the literature is controversial.^24,25^ This study did not find any association between depression and individual types of hormonal and non-hormonal contraception and therefore does not support a link between hormonal contraception and depression as one would expect more women using hormonal contraception compared to other forms of contraception to have a diagnosis of depression if hormonal contraception caused depression. The number of women using each type of contraceptive was, however, small and this limited the ability to generalise findings outside of this study. Level of functional impairment may differ between participants with different diagnoses, with some studies suggesting that psychotic illness presents with more severe cognitive and functional impairment than mood disorders such as depression.^26^ Cognitive impairment in people with MI has been associated with poor treatment adherence and it is possible that women with depression in this study were more likely to engage in contraceptive use because of differences in cognitive functioning compared to women with other MI.^27^ All women were screened for capacity to participate, but in-depth analysis of functionality was not part of the study.

There were no associations between medication and contraceptive use; however, more women using mood stabilisers used non-hormonal contraception as opposed to hormonal contraception. This study was unable to comment on why women on mood stabilisers preferentially used non-hormonal contraception; however, a possible explanation may have concerns regarding potential effects on mood as previously discussed.^24,25^ Teratogen use was common, with 41.8% (*n* = 79) of women prescribed a teratogen; however, teratogen prescription did not influence contraception usage (Fisher’s exact test, *p* = 0.531) (Table 2). International guidelines recommend that women using teratogenic medication receive FPE and some teratogenic medication, such as valproate, is contraindicated unless women have had FPE as part of the valproate pregnancy prevention plan.^20^ Despite guidelines recommending contraception in women of childbearing age who use valproate, less than half of women taking valproate (41.2%, *n* = 28) used contraception in this study. Additionally, 64.3% (*n* = 9) of women using lamotrigine; 40% (*n* = 4) of women using lithium; and no women using carbamazepine used contraception (Table 2). Importantly, 17.5% (*n* = 33) of the total sample were women who were using teratogenic medications and desired future pregnancies and while one woman using teratogenic medication was unsure if she wanted children. This equated to 41.8% of all women who were using teratogenic medication who wanted to fall pregnant in the future.

These findings are concerning as they reflect that a high percentage of women with MI may potentially expose their unborn child to teratogenic medication if they fall pregnant unintentionally. While prescribing guidelines recommend that all women with MI should be administered FPE, this becomes especially important when women with MI are using a teratogenic medication as this may influence pregnancy intent, timing of pregnancy, and decisions around psychiatric medication choice.^28^

### Family planning education in women with mental illness

Only a quarter, 26.8% (*n* = 51), of participants had FPE done by MHCPs. The low level of FPE was consistent with international literature.^15,16^ Family planning education was not associated with women being prescribed teratogens in this study, with only 27.8% (22 of the 79 women using teratogenic medication) having received FPE (*p* = 0.740). Although there was no association between receiving FPE and using contraception, decent quality FPE should increase uptake of contraceptive use, especially among women prescribed a known teratogen. The absence of an association between FPE and contraceptive use may be indicative of the quality of FPE dispensed. Data in this study were collected during 2016 and 2017. Changes occurred in 2017 to prescribing guidelines regarding sodium valproate which stipulated that valproate may not be prescribed to women of childbearing age unless pregnancy has been excluded and they are part of a pregnancy prevention programme.^20^ This study echoes the importance of these changes in the guidelines. The low levels of FPE, along with the lack of association between receiving FPE and contraceptive use in this study represent lost opportunities by MHCPs. Prospective studies may be better suited to determine the relationship between FPE and subsequent contraceptive choices in women with MI, especially those prescribed a teratogen such as valproate.

Women who were employed were more likely to have received FPE. As we have previously discussed when noting the association between education and barrier contraception use above, factors empowering women are associated with increased utilisation of FPE.^23^ This links back to the association between MI and poor socioeconomic circumstances and disempowerment of women with MI.^2,3,4,5,6^ This highlights a need for interventions that are targeted at empowering women with MI in order to allow them improved reproductive autonomy.

## Limitations

There were several limitations to this study. Convenience sampling was used and women were selected based on accessibility and thus the study may not be representative of the broader population. It is therefore not possible to generalise the results to the general population. A cross-sectional design was used and thus only associations, not causality may be assessed. Diagnosis was obtained from clinical diagnosis in the file and relied on the treating clinician’s diagnoses. The study was self-reported and subject to the accuracy of patients’ reporting. There may have been social desirability bias or recall bias. Although the study was anonymous, women with MI may still have given answers they perceived as being desired by the researcher as socially acceptable or been influenced by self-stigma around what they perceived to be socially acceptable. Women with MI may also have forgotten or incorrectly recalled some information.

This study did not examine patient life circumstances, contraceptive preferences, and beliefs and experiences of contraception. Other important social and demographic factors that may be linked to contraceptive use were not examined; for example, number of children in the household, household income, and financial stability. Having one or more children has been previously associated with increased usage of contraception.^5^ A history of postpartum onset of MI was not investigated. This study did not establish chronology of presentation of depressive symptoms and contraceptive use and it is therefore beyond this study to establish causality with regards to depression and contraceptive use. This study did not explore perceptions around STIs and use of barrier methods as a means of preventing STIs.

This study relied on quantitative data making use of a questionnaire. Qualitative data, such as in-depth interviews, would provide a deeper understanding of women with MI’s lived experience and attitude and beliefs towards contraception and family planning.

Similarly, this study was able to provide a preliminary ‘snapshot’ of reasons why women with MI do not use contraception; however, these reasons are limited by the quantitative nature of this study. This study does not delve into deeper reasons for non-use of contraception nor does it suggest ways to improve use.

Various studies examining contraceptive use in women with MI and the general population varied in their definition of contraception and it was difficult to draw comparisons between our results and the overall literature given these inconsistencies.

Lastly, doctors treating patients in this study were aware of the study and this may have influenced results as doctors may have unintentionally altered FPE practices as the study may have served to increase awareness around FPE.

## Conclusion

The prevalence of contraception in women with MI is similar to that of the general population; however, less than half of women used contraception consistently. Barrier methods were the most commonly used contraception. Only a quarter of women had received FPE. Women who were single were less likely to use contraception; however, being single was associated with use of barrier methods of contraception. Women with MI may have a perception that they are not at risk for an unplanned pregnancy if they are single, despite some reporting that they are sexually active but not in a relationship. This was supported by the lack of a partner being the commonest reason women did not use contraception. Women with depression were more likely to use contraception than women with other MI and a higher level of education was associated with the use of barrier methods of contraception. Women who were employed were more likely to have received FPE. Teratogen use was not associated with having received FPE or contraceptive use.

Importantly, this study demonstrated a high uptake of barrier methods of contraception compared to other countries. This suggests that women with MI in this study may be more protected from acquisition of STIs compared to other countries. Depot contraception was the second most common choice of contraception. Depot hormonal contraception is well-suited to women with MI as it reduces pill burden and women do not have to remember to take it daily, thus improving adherence. While barrier methods require negotiation of use with partners at every sexual encounter, depot contraception does not and may give women with MI more autonomy over their reproductive choices. Ideally, women with MI should be using barrier methods in addition to depot contraception for pregnancy prevention and STI prevention.

More studies are needed to understand women with MI’s attitudes, beliefs, and knowledge around contraception and family planning as well as to understand their individual reproductive needs. Larger studies are needed to explore individual contraceptive preferences and longitudinal data are needed to explore causality and associations between depression and contraceptive use.

## Contribution

This study emphasises the need for improved family planning services for women with MI. Only a quarter of women with MI have had FPE, despite FPE being important for allowing women with MI to make informed choices around contraception and planning future pregnancy. Factors that empower women with MI, such as higher level of education and employment, may improve ability to access FPE and contraception. This study suggests that barrier methods and depot hormonal contraception are commonly used and acceptable means of contraception for women with MI in South Africa. This study provides preliminary data that will help guide researchers with further in-depth qualitative studies that may address family planning needs in women with MI.
